# Baseline Values of Left Ventricular Systolic Function in Preterm Infants With Septic Shock: A Prospective Observational Study

**DOI:** 10.3389/fped.2022.839057

**Published:** 2022-03-29

**Authors:** Junjuan Zhong, Chun Shuai, Yue Wang, Jing Mo, Dongju Ma, Jing Zhang, Yingyi Lin, Jie Yang, Xiuzhen Ye

**Affiliations:** Neonatal Intensive Care Unit, Guangdong Women‘s and Children‘s Hospital, Guangzhou, China

**Keywords:** septic shock, preterm infants, stroke index, cardiac index, functional echocardiography

## Abstract

**Background and Aim:**

Guidelines recommended that cardiac index (CI) of term infants with septic shock should reach the target level of 3.3–6.0L/(min⋅m^2^). However, there are still no standard values for preterm infants with septic shock. Herein, we investigated the functional echocardiographic baseline values of left ventricular (LV) systolic functional parameters at the onset of septic shock in preterm infants and possible correlations between baseline values and poor outcomes.

**Materials and Methods:**

This was a prospective, observational, and longitudinal single-center study. Eligible infants were monitored for LV systolic functional parameters using functional echocardiography at the onset of septic shock. The primary study outcome was the difference in the baseline value of LV systolic functional parameters in preterm infants with septic shock with different gestational age (GA) and birth weight (BW). The secondary outcome was septic shock-associated death or severe brain injury (including grade 3–4 intraventricular hemorrhage or periventricular leukomalacia).

**Results:**

In total, 43 subjects met the criteria, with a median GA of 32^1/7^ weeks and BW of 1800 grams. No difference was observed in baseline values of LV systolic functional parameters among infants with different GA and BW. Infants were assigned to good and poor outcomes groups based on septic shock-associated death or severe brain injury. Out of 43 infants, 29 (67.4%) had good outcomes vs. 14 (32.6%) with poor outcomes. Stroke index (SI) [18.2 (11.1, 18.9) mL/m^2^ vs. 23.5 (18.9, 25.8) mL/m^2^, *p* = 0.017] and cardiac index (CI)[2.7 (1.6, 3.5) L/(min⋅m^2^) vs. 3.4 (3.0, 4.8) L/(min⋅m^2^), *p* = 0.015] in infants with poor outcomes were significantly lower (*P* < 0.05). Receiver operating characteristic (ROC) curve analysis showed that the cut-off values of SI and CI for predicting poor outcomes in preterm infants with septic shock were 19.5 mL/m^2^ (sensitivity, 73.9%; specificity, 81.8%) and 2.9L/(min⋅m^2^) (sensitivity, 78.3%; specificity, 72.7%), with area under the ROC curve (AUC) value of 0.755 and 0.759, respectively.

**Conclusion:**

There were no differences in baseline LV systolic functional values among preterm infants with septic shock with different GA and BW. However, preterm infants with SI<19.5mL/m^2^ and/or CI<2.9L/(min⋅m^2^) at the onset of septic shock were at high risk of having poor outcomes.

## Introduction

Sepsis is the leading cause of death for hospitalized infants, accounting for 45% of deaths in the neonatal intensive care unit (NICU) (1). Moreover, the sepsis-related mortality rate was reported to be twice as high among patients who developed cardiovascular dysfunction and septic shock (2). Hence, a precise assessment of cardiovascular compromise is essential for the appropriate management of infants with shock.

Heart rate, acid-base state, and lactate levels are the most common traditional indicators for the prediction of poor outcomes in infants with shock. However, recent studies on using color Doppler ultrasonography and near-infrared spectroscopy found that these indicators are relatively insensitive to tissue perfusion and oxygen delivery (3). In infants with shock, the imbalance between oxygen delivery and oxygen consumption can lead to metabolic disorders, organ dysfunction, and death (4). Functional echocardiographic analysis can be used to assess hemodynamics in neonates, provide a better understanding of the pathophysiological processes, and help monitor the effectiveness of and response to treatment decisions (5). In addition, left ventricular (LV) systolic functional parameters, such as stroke volume (SV) and cardiac output (CO), are essential for organs perfusion and oxygen delivery in critically ill infants with cardiovascular compromise (6, 7). Therefore, determining whether LV systolic function is normal or reduced is valuable in assessing perfusion, elucidating the mechanisms underlying hemodynamic instability, and helping target therapeutic strategies.

Guidelines recommended that cardiac index (CI) of in term infants with septic shock should reach the target level of 3.3–6.0L/(min⋅m^2^) (8). However, there are still no standard values for preterm infants with septic shock. Moreover, the diagnosis and treatment of septic shock in preterm infants need to be further improved.

We hypothesized that LVCO is reduced in preterm infants with poor outcomes compared to those with good outcomes at the onset of septic shock. We performed a prospective observational study to assess the baseline value of LV systolic functional parameters at the onset of septic shock in preterm infants using functional echocardiography and analyze the association with baseline value and poor outcomes.

## Methodology

This prospective observational study was conducted in an 80-bed tertiary level NICU, Guangdong Women‘s and Children‘s Hospital, Guangzhou, China, from 1 June 2019 to 30 November 2020. The study was approved by the Ethics Committee of the hospital, and informed consent was provided by all parents/guardians.

Clinical data were extracted from the electronic medical record. The first co-author performed all functional echocardiograms, after which all data were instantaneously revised by the second co-author, using an M9 ultrasound scanner with a 12-MHz transducer (Mindray, Shenzhen, China).

Left ventricular systolic functional parameters were measured by functional echocardiography during the infusion of saline bolus but before giving vasoactive drugs for all enrolled preterm infants. This period of time was considered as the onset of septic shock. All infants were clinically followed until 28 days of age. Infants were categorized in two cohorts based on septic shock-associated death or severe brain injury [(including grade 3–4 intraventricular hemorrhage (IVH) or periventricular leukomalacia (PVL)]: good and poor outcomes.

We included preterm infants with septic shock who were admitted to the NICU and underwent functional echocardiographic measurements at the onset of septic shock. Neonatal septic shock was defined based on the American College of Critical Care Medicine and International Pediatric Sepsis Consensus (8, 9). Exclusion criteria included multiple congenital anomalies, chromosomal abnormalities, congenital cardiac malformation (except non-hemodynamically significant patent ductus arteriosus and patent foramen ovale), persistent pulmonary hypertension, and suspected metabolic disorders.

Left ventricular systolic function was assessed by SV, stroke index (SI), CO, CI, and LV ejection fraction (LVEF) (10). All enrolled infants were placed in the left lateral decubitus position for the examination. Echocardiographic measurements were the average of three to five consecutive cardiac cycles. SV was calculated by multiplying the velocity-time integral (VTI) with the area of the LV outflow tract (LVOT), using the following formula: π × (LVOT diameter/2)^2^ × VTI.

SI, CO, and CI were calculated using the following formula: SI = SV/body surface area, CO = SV × hear rate, CI = (SV × hear rate)/body surface area.

LV ejection fraction was calculated using the Teichholz method. In addition, data regarding infants‘s gestational age (GA), birth weight (BW), gender, mode of delivery, 1 and 5 min Apgar scores, and clinical hemodynamic parameters [including blood pressure (BP) and heart rate (HR)] at the onset of septic shock were collected.

Sample size estimation was done according to the previous literature. The mortality rate of very low birth weight infants with septic shock was 71% (11). The required sample size was 42 infants in two groups (good and poor outcomes) with a power of 80% and a level of significance of 5% (two-sided). Continuous variables were expressed as median (IQR), and categorical variables as a number of cases and percentages. Kruskal Wallis Test was used to evaluate differences in LV systolic function measures in the three gestational age-stratified groups and birth weight-stratified groups at the onset of septic shock, respectively. The Mann-Whitney *U* test was used to evaluate differences in LV systolic function measures in infants with good and poor outcomes. Receiver operating characteristic (ROC) curve analysis was conducted to examine the ability of SI and CI to identify septic shock-associated death or severe brain injury, after which the sensitivity and specificity of optimal cut-offs were described. Data were analyzed using SPSS v. 23 (IBM, Armonk, NY). A *p* value<0.05 was considered statistically significant.

## Results

A total of 77 preterm infants were diagnosed with septic shock. According to the inclusion and exclusion criteria, 43 infants were included in the final analysis. Table 1 presents demographic data for the study population. Infants had median GA of 32^1/7^ weeks, and the median BW of 1800 g. There were more infants with GA in the range of 28^0/7^–33^6/7^ weeks (48.8%) and BW ≥ 1500 g (62.8%). The majority of the sample is appropriate for GA (small GA [n = 5], 11.6%). In addition, 30 out of 43 infants were male (69.8%).

**TABLE 1:** Demographic data in the studies patients.

	All infants
Number of infants	43
Gestational age (wk) median (IQR)	32^1/7^ (29^1/7^, 34^3/7^)
<28^0/7^wk, *n* (%)	7 (16.3)
28^0/7^–33^6/7^wk, *n* (%)	21 (48.8)
≥34^0/7^wk, *n* (%)	15 (34.9)
Birthweight (g) median (IQR)	1800 (1160, 2100)
<1000g, *n* (%)	6 (14.0)
1000–1499g, *n* (%)	10 (23.3)
≥1500g, *n* (%)	27 (62.8)
Small for gestational age, *n* (%)	5 (11.6)
Male sex, *n* (%)	30 (69.8)
Multiple births, *n* (%)	15 (34.9)
Cesarean section, *n* (%)	28 (65.1)
Apgar score median (IQR)	
1 min	8 (6, 9)
5 min	9 (9, 10)
Age at onset of septic shock (days) median (IQR)	2 (1, 2)
Late-onset sepsis, *n* (%)	6 (14.0)
Culture proven, *n* (%)	18 (41.9)

*IQR, interquartile range.*

Subgroup analyses were performed based on GA (<28^0/7^ weeks, 28^0/7^–33^6/7^ weeks, ≥ 34^0/7^ weeks) and BW (<1000g, 1000–1499g, ≥ 1500g). The baseline values of LV systolic functional parameters in preterm infants with septic shock at different GA- and BW-stratified distributions are reported as the median (IQR) in Tables 2, 3, respectively. The median of SI and CI at the onset of septic shock in these subgroups were below normal ranges (8). Yet, the baseline values of LV systolic functional parameters of these subgroups were not significantly different (P > 0.05).

**TABLE 2:** The baseline values of LV systolic functional parameters in preterm infants with septic shock at the different gestational age-stratified distribution.

	<28^0/7^w	28^0/7^–33^6/7^w	≥34^0/7^	*P*-value
Number of infants	7	21	15	
SV (mL/kg) median (IQR)	2.5 (2.0, 3.2)	2.2 (1.8, 2.6)	2.2 (1.5, 4.6)	0.439
SI (mL/m^2^) median (IQR)	18.1 (16.7, 20.4)	20.9 (18.2, 23.8)	28.3 (18.8, 55.4)	0.372
CO [mL/(kg⋅min)] median (IQR)	373 (278, 513)	368 (274, 419)	204 (165, 565)	0.385
CI [L/(min⋅m^2^)] median (IQR)	2.8 (2.4, 3.2)	3.4 (3.2, 3.6)	2.6 (2.0, 6.8)	0.385
CI<3.3L/(min⋅m^2^), n (%)	5 (71.4)	14 (66.7)	9 (60.0)	0.915
LVEF (%) median (IQR)	79 (68, 81)	69 (62, 72)	73 (67, 76)	0.352

*SV, stroke volume; SI, stroke index; CO, cardiac output; CI, cardiac index; LVEF, left ventricular ejection fraction.*

**TABLE 3:** The baseline values of LV systolic functional parameters in preterm infants with septic shock at the different birth weight-stratified distribution.

	<1000g	1000–1499g	≥1500g	*P* value
Number of infants	6	10	27	
SV (mL/kg) median (IQR)	2.6 (1.6, 3.5)	2.5 (2.2, 4.0)	1.8 (1.2, 2.2)	0.208
SI (mL/m^2^) median (IQR)	17.5 (16.4, 21.9)	21.2 (18.9, 32.4)	20.9 (12.6, 26.0)	0.651
CO [mL/(kg⋅min)] median (IQR)	401 (219, 560)	396 (373, 580)	250 (182, 296)	0.131
CI [L/(min⋅m^2^)] median (IQR)	2.7 (2.3, 3.5)	3.5 (3.2, 4.7)	2.6 (2.1, 3.3)	0.582
CI<3.3L/(min⋅m^2^), *n* (%)	4 (66.7)	5 (50.0)	19 (70.4)	0.476
LVEF (%) median (IQR)	76 (63, 80)	72 (69, 85)	63 (56, 73)	0.106

*SV, stroke volume; SI, stroke index; CO, cardiac output; CI, cardiac index; LVEF, left ventricular ejection fraction.*

The baseline values of LV systolic functional parameters of all included infants are summarized in Table 4. Twenty-nine infants (67.4%) had good outcomes, and 14 (32.6%) had poor outcomes. Among infants with poor outcomes, severe brain injury was found in 9 (20.9%), septic shock-associated death in 7 (16.3%) cases, and all-cause death within 28 days in 13 (30.2%) patients. Infants with poor outcomes had lower SI [18.2 (11.1, 18.9) mL/m^2^ vs. 23.5 (18.9, 25.8) mL/m^2^, *p* = 0.017] and lower CI [2.7 (1.6, 3.5) L/(min⋅m^2^) vs. 3.4 (3.0, 4.8) L/(min⋅m^2^), *p* = 0.015]. There were no differences in other baseline parameters between infants with good and poor outcomes, including BP, HR, SV, CO, and LVEF (P > 0.05).

**TABLE 4:** Comparison of baseline parameters in infants with good and poor outcomes.

	All	Good outcomes	Poor outcomes	*P*-value
Number of infants	43	29	14	
Gestational age (wk) median (IQR)	32^1/7^ (29^1/7^, 34^3/7^)	32^4/7^ (29^0/7,^ 34^1/7^)	30^2/7^ (29^1/7^, 35^6/7^)	0.805
Birthweight (g) median (IQR)	1800 (1160, 2100)	1820 (1190, 2125)	1580 (1128, 2153)	0.907
MAP (mm Hg) median (IQR)	29 (27, 33)	29 (28, 32)	27 (22, 33)	0.296
SBP (mm Hg) median (IQR)	46 (40, 48)	46 (40, 50)	45 (37, 49)	0.835
DBP (mm Hg) median (IQR)	23 (20, 25)	24 (22, 24)	21 (17, 25)	0.336
HR (bpm) median (IQR)	145 (140, 156)	145 (136, 156)	149 (141, 170)	0.277
SV (mL/kg) median (IQR)	2.2 (1.5, 3.6)	2.8 (2.0, 4.8)	2.1 (0.7, 2.5)	0.145
SI (mL/m^2^) median (IQR)	20.8 (16.4, 29.5)	23.5 (18.9, 35.8)	18.2 (11.1, 18.9)	0.017
CO [mL/(kg⋅min)] median (IQR)	267 (213, 466)	466 (296, 648)	352 (149, 407)	0.221
CI [L/(min⋅m^2^)] median (IQR)	2.9 (2.3, 3.6)	3.4 (3.0, 4.8)	2.7 (1.6, 3.5)	0.015
CI<3.3L/(min⋅m^2^), *n* (%)	28 (65.1)	16 (55.2)	12 (85.7)	0.086
LVEF (%) median (IQR)	69 (62, 78)	72 (66, 76)	76 (62, 84)	0.931

*MAP, mean arterial pressure; SBP, systolic blood pressure; DBP, diastolic blood pressure; HR, heart rate; SV, stroke volume; SI, stroke index; CO, cardiac output; CI, cardiac index; LVEF, left ventricular ejection fraction.*

**FIGURE 1:**
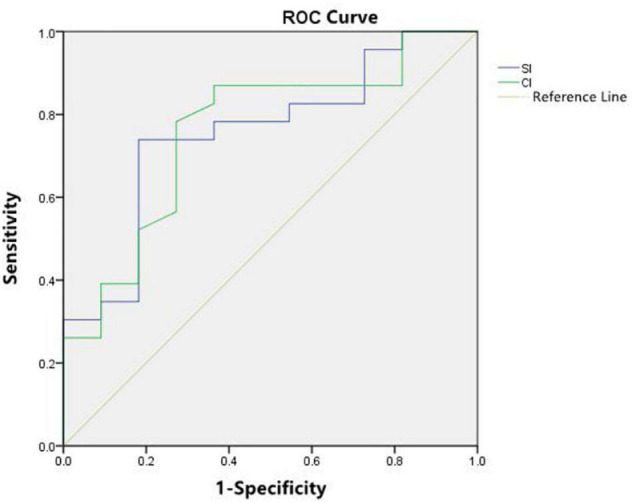
Receiver operating characteristic (ROC) curve analysis showed good performance of stroke index (SI) and cardiac index (CI) as predictors of subsequent septic shock-related death and severe brain injury.

The area under the curve (AUC) for SI to identify poor outcomes was 0.755 (95% CI 0.582, 0.928), and for CI was 0.759 (95% CI 0.583, 0.928) (Figure 1). The sensitivity and specificity of optimal cut-offs for SI and CI for poor outcomes are summarized in Table 5.

**TABLE 5:** Value of SI and CI in predicting septic shock related-death or severe brain injury.

	SI	CI
Cut-off	19.5	2.9
AUC	0.755 (95.0% CI: 0.582–0.928)	0.759 (95.0% CI: 0.583–0.928)
*P*	0.018	0.016
Sensitivity	73.9%	78.3%
Specificity	81.8%	72.7%

*SI, stroke index; CI, cardiac index; AUC, area under the curve.*

## Discussion

To the best of our knowledge, this is the first study that described echocardiographic LV systolic functional measurements at the onset of septic shock in preterm infants. This study also explored the value of baseline parameters in predicting the prognosis of preterm infants with septic shock. The key findings of this study are follo wing: (a) low SI and/or low CI at the onset of septic shock were correlated with the risk of poor prognosis in preterm infants, and the predicting value of SI and CI was similar; (b) isolated low BP, low SV, low CO or high HR values at the onset of septic shock cannot clearly identify the risk of poor outcomes; (c) there was no significant difference of baseline value of LV systolic functional parameters among preterm infants with different GA and BW. Using ROC analyses, we determined that the recorded baseline SI and CI were associated with septic shock-associated death and/or severe brain injury in preterm infants. SI ≥ 19.5mL/m^2^ and/or CI ≥ 2.9L/(min⋅m^2^) was correlated with the lowest rates of septic shock-associated death and/or severe brain injury.

The mortality rate of preterm infants with septic shock in our study (16.3% [7/43]) was significantly lower than that reported in the previous study (40–71%) (12), which is probably related to the use of functional echocardiographic techniques in assessment of LV cardiac output that can better guide management regimes for preterm infants with septic shock. LVCO is an important hemodynamic parameter that aids in targeting organ perfusion and oxygen delivery in infants with cardiovascular compromise. The latest guideline recommends using both transthoracic echocardiography and/or transesophageal echocardiography for the assessment of LVCO in evaluating responses to treatment (13). Thus, the reliability of subjective assessment of LVCO is questionable.

Clinical research on outcomes in neonates with septic shock has been mainly focusing on blood pressure, while the utility of hemodynamic parameters as clinical surrogates of hemodynamic status is not fully understood. The present study showed no significant difference in baseline blood pressure at the onset of septic shock between infants with good and poor outcomes. Therefore, the use of blood pressure to assess hemodynamic status remains controversial. A recent study found that by using third percentile cut-off for systolic BP, which is a common practice in the NICU, a large majority of infants with low CO fail to be detected (14). This evidently shows that BP values and LVCO are weakly correlated regardless of GA and underlying etiology. Moreover, some studies failed to show an improved prognosis after the treatment of hypotension based on mean arterial BP thresholds (15). The uncertainty about the lower limit of normal BP range in preterm infants is even more challenging. Our results further confirmed previous observations suggesting that BP should not be used as an isolated surrogate of hemodynamic status in preterm infants, irrespective of the underlying pathology, gestational age, and postnatal age.

Our study found that low SI and/or low CI at the onset of septic shock were correlated with the risk of poor prognosis in preterm infants, which is inconsistent with prior study results (16, 17). Previous studies reported high cardiac output in those with early onset of septic shock, especially in non-surviving infants, which may be related to particularities of our subjects sample with early age of onset. In this study, the median age at onset was 2 days, while the study above reported an onset of sepsis at 4 days after birth. Also, low SI and/or CI at the onset of septic shock in preterm infants could be caused by different factors (18). First, the myocardium of the preterm infant possesses an inefficient contractile mechanism and a preponderance of non-contractile, poorly compliant collagen, leading to impaired diastolic performance, particularly during the early transitional period. Furthermore, preterm infants exhibit a high resting peripheral vascular tone owing to the relatively higher concentration of vasoconstrictive receptors at the expense of vasodilatory. Additionally, the myocardium, on the other hand, lacks adequate adrenergic innervation limiting its ability to increase contractility. Therefore, our results are credible and aligned with our expectations.

### Study Limitation

Our study has several limitations. First, we did not include a control group, which may lead to some bias. Nevertheless, hyper-metabolic diseases like shock require higher CO to satisfy the enhanced metabolic demand of the body. Therefore, comparisons of CO between normal and ill infants may not be significant. Second, our observations are limited to a relatively small sample size despite the apparent plausibility of low SI and low CI at the onset of septic shock in preterm infants with poor outcomes. Third, we assessed the single value of baseline parameters as predictors of poor outcomes without considering the duration of low SI and low CI or subsequent course of the preterm infants.

## Conclusion

Low SI and/or low CI at the onset of septic shock were associated with death and/or severe brain injury in preterm infants. Functional echocardiographic parameters used to determine the hemodynamic status of shock may be used to understand the pathophysiology and predict poor outcomes in preterm infants with septic shock to inform decision-making regarding indications, timing, and approach to therapy. Our results provided support for our hypothesis that the baseline value of LV systolic functional parameters could be used to predict poor outcomes in preterm infants with septic shock. Therefore, these findings deserve further investigation in larger samples and future studies to further verify their clinical significance.

## Data Availability Statement

The raw data supporting the conclusions of this article will be made available by the authors, without undue reservation.

## Ethics Statement

The studies involving human participants were reviewed and approved by the Ethical Committee (No. 202101188) in the Guangdong Women‘s and Children‘s Hospital. Written informed consent to participate in this study was provided by the participants’ legal guardian/next of kin.

## Author Contributions

XY, CS, YW, and JM conceptualized and designed the study, organized the acquisition of data, and reviewed and revised both the analyses and manuscript. JZo, JZa, DM, and YL drafted and critically evaluated the article. JY corrected the final version of the manuscript. All authors approved the final manuscript to be published.

## Conflict of Interest

The authors declare that the research was conducted in the absence of any commercial or financial relationships that could be construed as a potential conflict of interest.

## Publisher’s Note

All claims expressed in this article are solely those of the authors and do not necessarily represent those of their affiliated organizations, or those of the publisher, the editors and the reviewers. Any product that may be evaluated in this article, or claim that may be made by its manufacturer, is not guaranteed or endorsed by the publisher.
